# Contributions of T Lymphocyte Abnormalities to Therapeutic Outcomes in Newly Diagnosed Patients with Immune Thrombocytopenia

**DOI:** 10.1371/journal.pone.0126601

**Published:** 2015-05-15

**Authors:** Zhenhua Zhao, Lei Yang, Guohua Yang, Yun Zhuang, Xifeng Qian, Xin Zhou, Dajiang Xiao, Yunfeng Shen

**Affiliations:** 1 Department of Otolaryngology, Nanjing Medical University Affiliated Wuxi Second Hospital, Wuxi, Jiangsu, 214002, People's Republic of China; 2 Department of Hematology, Wuxi People’s Hospital Affiliated to Nanjing Medical University, Wuxi, Jiangsu, 214023, People's Republic of China; Institut National de la Santé et de la Recherche Médicale (INSERM), FRANCE

## Abstract

T cell abnormalities have been reported to play an important role in pathogenesis of immune thrombocytopenia (ITP) besides specific autoantibodies towards platelet. The aim of this study was to explore the clinical importance of T lymphocyte subsets in adult patients with newly diagnosed ITP before and after first-line treatment. Elderly ITP patients were also studied and we tried to analyze the relationships between these items and therapeutic outcomes. The patients were treated with intravenous immunoglobulin (IVIG) plus corticosteroids and therapeutic responses were evaluated. As a result, compared with the controls, absolute lymphocyte counts in ITP patients decreased significantly before treatment. After treatment, lymphocyte counts restored to control level regardless of their treatment outcomes. In addition, we observed increased IgG and CD19^+^ cell expression and decreased CD4^+^/CD8^+^ cell ratio in both whole ITP group and elderly group before treatment. After treatment, the increased IgG and CD19^+^ cell expression could be reduced in both respond and non-respond group regardless of patient age, while CD4^+^/CD8^+^ cell ratio could not be corrected in non-respond ITP patients. In non-respond ITP patients, increased CD8^+^ cell expression was noticed and could not be corrected by first-line treatment. Furthermore, even lower NK cell expression was found in non-respond elderly patients after treatment when compared with that in controls. Our findings suggest that ITP patients usually had less numbers of peripheral lymphocytes and patients with higher levels of CD8^+^ cells or lower levels of CD4^+^/CD8^+^ cell ratio were less likely to respond to first-line treatment. Lower levels of NK cells made therapies in elderly ITP patients even more difficult.

## Introduction

Immune thrombocytopenia (ITP) is an immune-mediated bleeding disorder in which platelets are opsonized by autoantibodies and destroyed by macrophages in the spleen[1–3]. Although autoantibodies mediated platelet destruction is considered to play a crucial role, increasing evidence suggests that the mechanism of ITP is complicated. Autoantibodies mediated dysmegakaryocytopoiesis may also contribute to thrombocytopenia[4–5]. In recent decade, T-cell-mediated immune abnormalities have been considered equally important in the pathogenesis of ITP. T cell abnormalities include a significant shift towards T helper (Th) 1 cells and Th17 pro-inflammatory immune responses[6–7], the decreased number or defective function of regulatory T cells (Treg)[8–9], and the platelet destruction by cytotoxic T lymphocytes (CTLs)[10–13]. There are also several reports of different amounts of natural killer (NK) cells that are nevertheless functionally defective in peripheral blood from ITP patients[14–16].

The incidence of ITP is thought to be higher in women of reproductive age. Recently, more and more epidemiological data have found that the incidence of ITP increases with age, especially in those elderly patients (ages ≥ 60)[17–18]. In elderly patients with ITP, most have chronic disease courses, more common hemorrhagic events, poor response to various treatment options and thus high mortality directly associated with disease itself[19–20]. Furthermore, clinicians need to pay much more attention to the treatment of elderly ITP patients.

Although ITP is well known nowadays and the first line treatments for ITP patients focus on corticosteroids and intravenous immunoglobulin(IVIG), the diagnosis is still exclusive and the therapies are non-specific. The phases, severity of bleeding, mortality and sensitivity to treatments vary widely in ITP patients[21–22], which makes the disease heterogeneous. Individualized treatments are urgent according to different disease mechanisms, different ages and maybe different clinical bleeding symptoms.

In this retrospective study, we investigated absolute lymphocyte counts, IgG levels and T lymphocyte subsets in peripheral blood of 61 adult patients with newly diagnosed ITP before and after first-line treatment. The situations of elderly patients were emphasized in our study and we tried to analyze the association between these items and therapeutic outcomes.

## Materials and Methods

### Patients and controls

We retrospectively reviewed 61 patients with newly diagnosed ITP(37 females and 24 males, 31 age<60 years and 30 age ≥60 years) between March 2012 and August 2013 at the Department of Hematology, Wuxi People’s Hospital Affiliated to Nanjing Medical University. These patients did not undergo any form of therapy before diagnosis (Table 1). The diagnosis in all patients, based on the criteria for newly diagnosed ITP as previously described[21], exhibited isolated thrombocytopenia within 3 months from diagnosis, normal or increased bone marrow megakaryocytes, normal spleen size, and no secondary immune or non-immune abnormality that could account for the thrombocytopenia. Patients with systemic lupus erythematosus and/or antiphospholipid syndrome were excluded from this study, as were pregnant patients or those with concomitant human immunodeficiency or hepatitis C virus infection. Median age (range) and median platelet count (range) at the time of enrollment were 54 years (18–87 years) and 9×10^9^/L(0–25×10^9^/L), respectively. Median age (range) and median platelet count (range) at the time of enrollment in elderly patients were 71 years (61–87 years) and 10×10^9^/L(0–23×10^9^/L), respectively. Control blood was obtained from 20 healthy blood donors (11 females and 9 males; age range, 22–78 years; median, 45 years) with no history of blood transfusions or pregnancies. Platelet counts ranged from 135 to 280×10^9^/L, with the median count of 205×10^9^/L.

**Table 1:** Patients with newly diagnosed ITP.

Patient	Age(y)	Sex	Platelet count(10^9^/L)	IgG(g/L)	Bleeding symptoms*	Treatment respond^#^	Salvage treatment^$^
1	57	F	10	23.1	PT/EC/GB/OBL/GUH	CR	—
2	87	M	10	18.7	PT	CR	—
3	61	M	18	19.8	PT/OBL	NR	VCR
4	61	F	9	17.8	PT/EC/OBL	NR	VCR/TPO
5	58	F	13	—	EC	CR	—
6	77	M	12	—	PT/EP	R	—
7	80	F	22	—	PT/EC	NR	VCR/R
8	69	M	18	23.5	PT/EC	NR	VCR/CsA
9	36	F	5	—	PT/EC/GB/GH	NR	VCR/TPO/IL-11
10	32	M	4	18.3	PT/EP	R	—
11	77	M	9	—	—	R	—
12	47	F	8	18.1	PT/EC	NR	AZT
13	47	F	3	19.1	PT/EC/OBL	CR	—
14	62	F	10	29.2	PT/EP/GB	R	—
15	24	F	6	—	PT/EC/EP/OBL	CR	—
16	18	F	8	—	PT/EC	CR	—
17	54	F	19	—	PT/EC	R	—
18	58	F	2	—	—	NR	AZT/IL-11
19	24	F	5	20	PT/EC	CR	—
20	64	F	11	—	PT/EC	R	—
21	33	F	5	29.5	PT/EC	CR	—
22	57	F	11	26.3	PT/EC/GB	R	—
23	79	M	11	25.9	PT/EC/OBL	CR	—
24	65	F	21	19.5	EC	NR	DAN
25	68	F	3	17.7	PT/EC/GB	CR	—
26	65	M	15	23	EC	R	—
27	20	F	2	—	PT/EC	CR	—
28	35	F	8	18.2	GB	NR	VCR/TPO
29	85	F	6	20.8	PT/EC	R	—
30	79	M	4	18.5	PT/EC/OBL	NR	R
31	69	M	23	—	PT/OBL	R	—
32	50	F	2	18.3	PT/EC	R	—
33^&^	67	M	7	—	PT/EC	NR	VCR/TPO/R
34	72	F	9	—	—	R	—
35	68	F	5	27.1	EC/GB	NR	VCR/AZT
36	75	M	11	—	—	R	—
37	32	M	6	19.2	PT/EC/GB/OBL	CR	—
38	74	F	3	30.1	PT/EC/OBL	CR	—
39	80	F	1	27.7	PT/EC	R	—
40	61	M	13	—	EP	R	—
41	53	M	10	—	PT/EC/GB	R	—
42	74	M	15	—	PT/EC	NR	VCR
43	70	M	5	24.7	PT/EC/OBL	CR	—
44	26	F	5	20	PT/EC/GH	CR	—
45	34	F	18	22.3	PT/EC	R	—
46	20	F	3	25.9	PT/EC	CR	—
47	22	M	5	19.9	PT/EC/OBL	NR	VCR/TPO
48	35	M	5	17.2	PT/EC	CR	—
49	25	F	2	31.4	PT/EC/GB	CR	—
50	73	F	0	17.8	EC/GB	R	—
51	26	F	13	21.5	PT/EC	CR	—
52	46	F	20	—	PT/EP/GB	CR	—
53	38	F	5	—	PT/EC/OBL	CR	—
54	56	M	9	16.4	EP/GB	R	—
55	45	M	25	11	PT/EC	NR	VCR
56	44	F	14	23	PT/EC/OBL	R	—
57	74	F	9	19.2	PT/EC/EP/OBL	R	—
58	65	F	2	25.3	PT/EC	NR	VCR/TPO
59	61	M	12	21.1	GB/OBL	R	—
60	70	M	16	15.8	PT/EC/GB/OBL	NR	R
61	32	M	10	12.6	EP/GB	CR	—

* PT, petechiae; EC, ecchymoses; EP, epistaxis; GUH, genitourinary hemorrhage; GH, gingival hemorrhage; GB, gums bleeding; OBL, oral blood blister.

# CR, complete response; R, response; NR, no response.

$ VCR, vincristin; R, rituximab; AZT, azathioprine; DAN, danazol; CsA, cyclosporine; IL-11, interleukin-11.

& The patient died of cerebral hemorrhage.

### Treatment

Patients with newly diagnosed ITP (platelet counts less than 30×10^9^/L and/or with bleeding) in this retrospective study were all treated with IVIG (400 mg/kg per day for 5 days) plus corticosteroids(most frequently prednisone at 1.0 mg/kg per day for 1–3 weeks, then tapered) according to first-line treatment standardization[22]. Some patients with severe bleeding were also given platelet transfusion in order to quickly increase platelet counts. In addition to standard therapy, vincristine, rituximab, azathioprine, danazol, cyclosporine, interleukin-11 and TPO were used as salvage therapy on non-respond patients.

### Treatment responses and follow-up

Treatment responses were evaluated according to the consensus definition of the International Working Group[21]. “Complete response” (CR) was defined as a platelet count equal to or greater than 100×10^9^/L and absence of bleeding. “Response” (R) was defined as a platelet count equal to or greater than 30×10^9^/L and at least 2-fold increase the baseline count and absence of bleeding, and “no response” (NR) was defined as a platelet count lower than 30×10^9^/L or less than 2-fold increase of baseline platelet count or bleeding. For each patient, the observation period started on the day of the initial diagnosis. Patients were followed until the observation period ended on August 30, 2013or until death (due to any cause) if it occurred before this date.

### Statistical analysis

This study was approved by ethics committee of Wuxi People’s Hospital. In this study, we analyzed lymphocyte numbers, IgG levels and T lymphocyte subsets in ITP patients before and seven days after treatment. Since this is a retrospective study, all data including those of control samples were identified from the hospital’s database and analyzed anonymously, no consent was given. We checked all the items on the STROBE checklist of case-control studies as well (S1 STROBE Checklist). All data are presented as mean plus or minus standard deviation (SD). Categorical variables between groups were compared using the Chi-square test. Quantitative values were analyzed by the One-way ANOVA test of parametric data and Kruskal-Wallis test of nonparametric data. Paired Student’s t test was used to assess values of ITP patients before and after treatment. A P value of <0.05 was regarded as statistically significant in two-sided tests. Data were analyzed using SPSS statistical software version 18 (SPSS, Chicago, IL, USA).

## Results

### Responses to first-line treatment

Of the 61 patients included in this retrospective study, 44 (72.13%) displayed CR or R, and 17(27.87%) displayed NR to treatment. Of the 30elderly patients (age ≥60 years), 19 (63.33%) displayed CR or R, and 11(36.67%) displayed NR to treatment. There was no significant difference in the response rate between the whole patient group and the elderly group (p = 0.47). Although skin bleeding, oral blood blister, epistaxis and gingival bleeding were frequently seen in our ITP patients, severe bleeding events, especially life-threatening ones, were much less common. Among the seventeen NR patients, five became chronic. And of these five patients, three were old patients with low platelet count but no severe bleeding at the time of diagnosis. One elderly patient who resisted to the first-line treatment became refractory after salvage treatments and died of cerebral hemorrhage in the end (Table 1).

### Absolute lymphocyte counts in different analyzing groups before and after treatment

**Fig 1:**
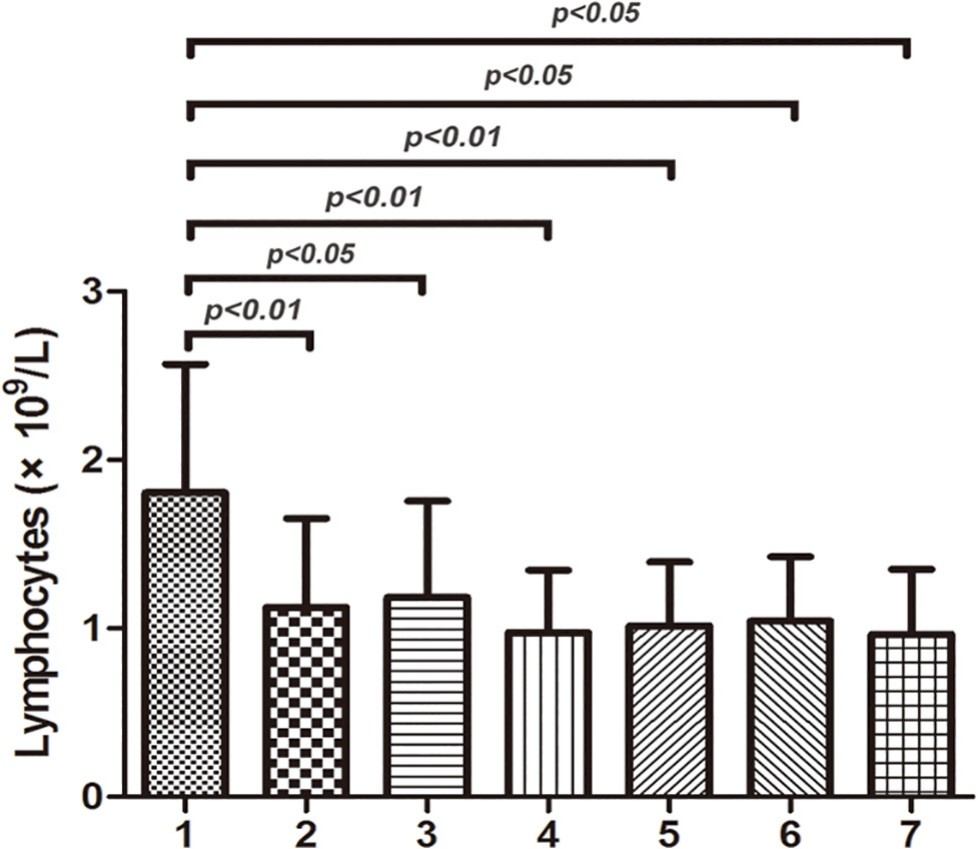
Absolute lymphocyte counts in different analyzing groups before treatment. Absolute lymphocyte counts (ALC) in group1 (control group), group2 (ITP group), group3 (respond ITP group), group4 (non-respond ITP group), group5 (elderly ITP group), group6 (respond elderly ITP group), group7 (non-respond elderly ITP group) were 1.81±0.76×10^9^/L, 1.13±0.53×10^9^/L, 1.19±0.57×10^9^/L, 0.98±0.37×10^9^/L, 1.02±0.38×10^9^/L, 1.04±0.38×10^9^/L, 0.97±0.38×10^9^/L, respectively. Regardless of the treatment outcomes and patient age, ALC in ITP groups were much lower than that in controls before treatment (P<0.05).

Compared with absolute lymphocyte counts (ALC) in the control group (mean±SD, 1.81±0.76×10^9^/L), ALC in ITP group (1.13±0.53×10^9^/L, P<0.01) was much lower before treatment, and so was that number in elderly ITP patients (1.02±0.38×10^9^/L, P<0.01). Regardless of the treatment outcomes and patient age, ALC in ITP groups were much lower than that in controls before treatment (Fig 1). After first-line treatment, as shown in Table 2, ALC in ITP groups with different outcomes or ages increased and showed no significant differences with the controls.

**Table 2:** Absolute lymphocyte counts in different analyzing groups before and after treatment (×10^9^/L).

	ITP	ITP(CR)	ITP(NR)	Elderly ITP	Elderly ITP(CR)	Elderly ITP(NR)
Before treatment	1.13±0.53	1.19±0.57	0.98±0.37	1.02±0.38	1.04±0.38	0.97±0.38
After treatment	1.62±0.70^***^	1.74±0.73^***^	1.30±0.48^*^	1.49±0.57^***^	1.53±0.62^***^	1.42±0.50^*^

*** P<0.001 compared with that before treatment.

* P<0.05 compared with that before treatment.

### IgG and T lymphocyte subsets expression in different groups before treatment

**Fig 2:**
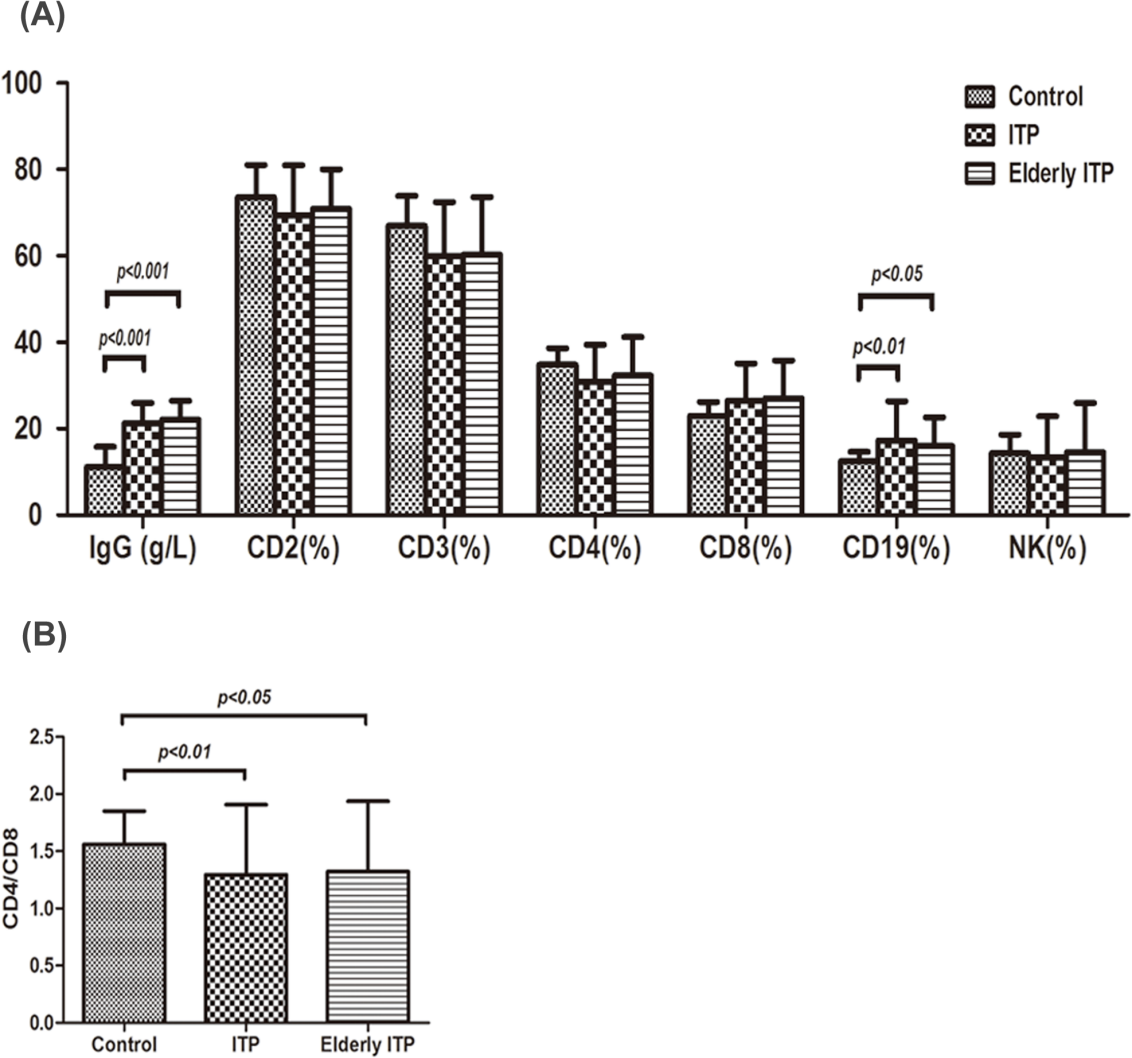
IgG and T lymphocyte subsets expression in different groups. (A) The levels of serum IgG (mean±SD, 21.33±4.64 g/L, P<0.001) and CD19^+^ cell percentage (17.27±9.14%, P<0.01) in ITP patients were higher than those in control group (11.15±4.61 g/L, 12.47±2.19%),respectively. And so did those in elderly ITP group when compared with the controls, which were 22.16±4.28 g/L (P<0.001) and 15.94±6.68% (P<0.05) respectively. Serum IgG and CD19^+^ cell percentage did not differ significantly between the whole ITP patients and the elderly ITP patients. There were no remarkable differences in CD2^+^, CD3^+^,CD4^+^, CD8^+^ and NK cell expression among the three groups. (B) A significant reduction in CD4^+^/CD8^+^ cell ratio was seen in both ITP group (1.29±0.61, P<0.01) and elderly ITP group (1.32±0.62, P<0.05) compared with controls(1.56±0.29). CD4^+^/CD8^+^ cell ratio did not differ significantly between the ITP patients and the elderly ITP patients.

The levels of serum IgG (21.33±4.64 g/L, P<0.001) and CD19^+^ cell percentage(17.27±9.14%, P<0.01) in ITP patients were higher than those in control group (11.15±4.61 g/L, 12.47±2.19%),respectively (Fig 2A). And so were those in elderly ITP group when compared with the controls, which were 22.16±4.28 g/L (P<0.001) and 15.94±6.68% (P<0.05) respectively. However, serum IgG and CD19^+^ cell percentage did not differ significantly between the ITP group and the elderly ITP group. A significant reduction in CD4^+^/CD8^+^ cell ratio was seen in both ITP group (1.29±0.61, P<0.01) and elderly ITP group (1.32±0.62, P<0.05) compared with controls(1.56±0.29, Fig 2B). CD4^+^/CD8^+^ cell ratio did not differ significantly between the ITP group and the elderly ITP group. CD2^+^, CD3^+^, CD4^+^, CD8^+^ and NK cell percentage showed no significant differences among three groups (Fig 2A).

### IgG and T lymphocyte subsets expression in different outcome groups of ITP patients before treatment

**Fig 3:**
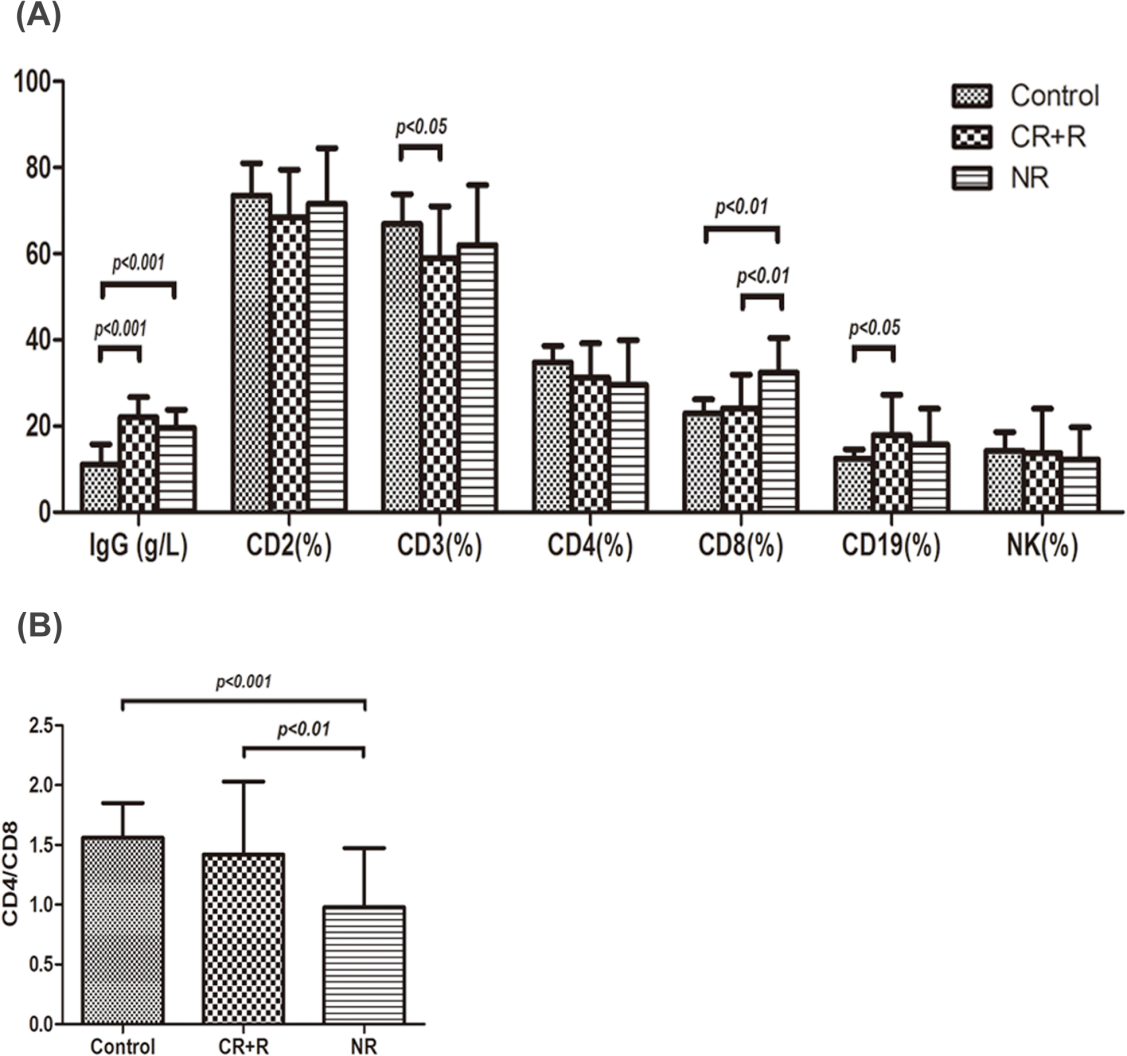
IgG and T lymphocyte subsets expression in different outcome groups of ITP patients. (A) IgG levels in healthy controls, respond group (CR+R) and non-respond group (NR) were 11.15±4.61g/L, 22.07±4.65 g/L, 19.54±4.28 g/L, respectively. IgG expression in both respond and non-respond groups were remarkably higher than that in controls (P<0.001), but there was no significant difference between the respond group and the non-respond group. CD3^+^ cell percentage in respond group was 59.00±12.08%, which was lower than that in controls (66.98±6.86%, P<0.05). CD19^+^ cell percentage in respond group was 17.88±9.45%, which was higher than that in controls (12.47±2.19%, P<0.05). Increased CD8^+^ cell expression was observed in non-respond group (32.42±8.07%, P<0.01) compared with that in controls (22.99±3.24%) and that in respond group (24.13±7.83%). There were no remarkable differences in CD2^+^, CD4^+^ and NK cell expression among the three groups. (B) A significant reduction in CD4^+^/CD8^+^ cell ratio was seen in non-respond group (0.98±0.50) compared with that in controls (1.56±0.29, P<0.001) and that in respond group (1.42±0.61, P<0.01).

As shown in Fig 3A, IgG levels in healthy controls, respond group (CR+R) and non-respond group (NR) were 11.15±4.61g/L, 22.07±4.65 g/L, 19.54±4.28 g/L, respectively. IgG expression in both respond and non-respond group were remarkably higher than that in controls before treatment (P<0.001), but there was no significant difference between respond and non-respond group. CD3^+^ cell percentage in respond group was 59.00±12.08%, which was lower than that in controls (66.98±6.86%, P<0.05). CD19^+^ cell percentage in respond group was 17.88±9.45%, which was higher than that in controls (12.47±2.19%, P<0.05). Increased CD8^+^ cell percentage was observed in non-respond group (32.42±8.07%, P<0.01) compared with that in controls (22.99±3.24%) and respond group (24.13±7.83%). A significant reduction in CD4^+^/CD8^+^ cell ratio was seen in non-respond group (0.98±0.50) compared with that in controls (1.56±0.29, P<0.001) and respond group (1.42±0.61, P<0.01), shown in Fig 3B.

### IgG and T lymphocyte subsets expression in different outcome groups of elderly ITP patients before treatment

**Fig 4:**
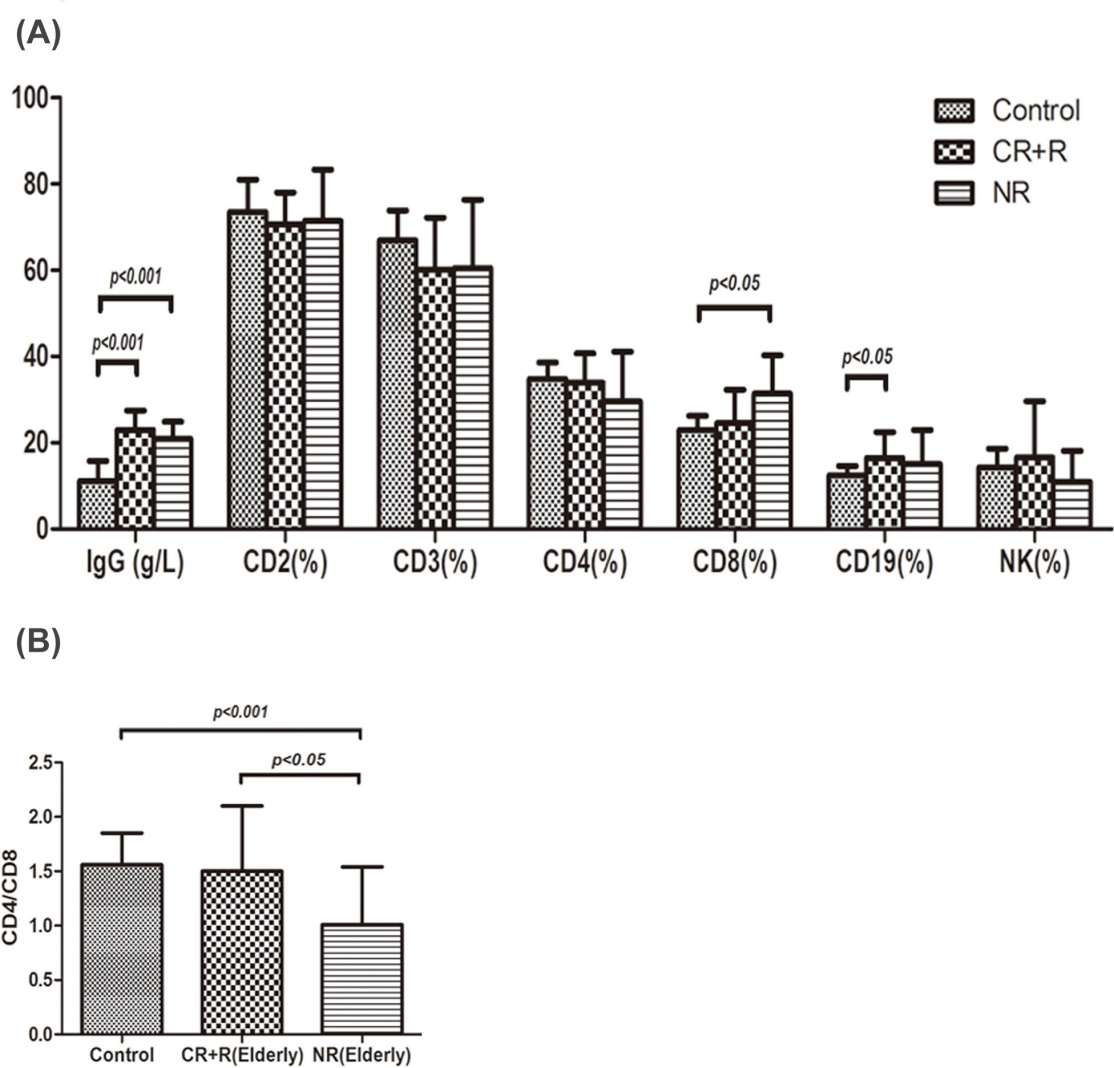
IgG and T lymphocyte subsets expression in different outcome groups of elderly ITP patients. (A) IgG levels in healthy controls, respond group (CR+R) and non-respond group (NR) were 11.15±4.61g/L, 22.99±4.46 g/L, 20.91±3.95 g/L, respectively. IgG expression in both respond and non-respond groups were remarkably higher than that in controls (P<0.001), but there was no significant difference between the respond group and the non-respond group. CD19^+^ cell percentage in respond group was 16.46±6.03%, which was higher than that in controls (12.47±2.19%, P<0.05). Increased CD8^+^ cell expression was observed in non-respond group (31.38±8.88%, P<0.05) compared with that in controls (22.99±3.24%). There were no remarkable differences in CD2^+^, CD3^+^,CD4^+^ and NK cell expression among the three groups. (B) A significant reduction in CD4^+^/CD8^+^ cell ratio was seen in non-respond group (1.01±0.53) compared with that in controls (1.56±0.29, P<0.001) and that in respond group (1.50±0.60, P<0.05).

As shown in Fig 4A, IgG levels in healthy controls, respond group (CR+R) and non-respond group (NR) were 11.15±4.61g/L, 22.99±4.46 g/L, 20.91±3.95 g/L, respectively. IgG expression in both respond and non-respond group was remarkably higher than that in controls (P<0.001), but there was no significant difference between respond and non-respond group. CD19^+^ cell percentage in respond group was 16.46±6.03%, which was higher than that in controls (12.47±2.19%, P<0.05). Increased CD8^+^ cell percentage was observed in non-respond group (31.38±8.88%, P<0.05) compared with that in controls (22.99±3.24%). A significant reduction in CD4^+^/CD8^+^ cell ratio was seen in non-respond group (1.01±0.53) compared with that in controls (1.56±0.29, P<0.001) and respond group (1.50±0.60, P<0.05), shown in Fig 4B. However, IgG and T lymphocyte subsets expression did not differ significantly between the whole ITP patients and the elderly patients in both respond and non-respond group before treatment.

### IgG and T lymphocyte subsets expression in both ITP and elderly ITP patients (CR+R) before and after first-line treatment

After treatment, the level of serum IgG (14.91±5.01 g/L, P<0.001) was much lower than that before treatment (22.07±4.65 g/L) in respond ITP group, but still higher than that in controls (11.15±4.61 g/L, P<0.05). CD3^+^ cell percentage in respond ITP group was 59.79±12.86%, which was still lower than that in controls (66.98±6.86%, P<0.05). CD19^+^percentage was 12.55±5.48%, which was lower than that before treatment (17.88±9.45%, P<0.001) and did not differ significantly from the controls (Table 3).

**Table 3:** IgG and T lymphocyte subsets expression in both ITP and elderly ITP patients (CR+R) before and after first-line treatment(mean±SD).

	IgG(g/L)	CD2(%)	CD3(%)	CD4(%)	CD8(%)	CD4/CD8	CD19(%)	NK(%)
Control	11.15±4.61	73.55±7.46	66.98±6.86	34.79±3.85	22.99±3.24	1.56±0.29	12.47±2.19	14.32±4.28
Before(ITP)	22.07±4.65	68.52±11.06	59.00±12.08	31.24±7.97	24.13±7.83	1.42±0.61	17.88±9.45	13.73±10.31
After(ITP)	14.91±5.01^***^ ^#^	68.30±10.39	59.79±12.86^#^	32.06±7.88	24.10±7.21	1.42±0.49	12.55±5.48^***^	14.04±8.85
Before(elderly)	22.99±4.46	70.61±7.45	60.18±12.04	33.85±6.86	24.55±7.75	1.50±0.60	16.46±6.03	16.57±13.08
After(elderly)	16.21±4.01^***^ ^#^	71.06±6.50	60.62±11.67	34.35±7.89	22.78±6.47	1.56±0.40	11.78±4.91^***^	17.72±11.49

*** P<0.001 compared with the group before treatment.

**#** P<0.05 compared with the control group.

After treatment, the level of serum IgG (16.21±4.01 g/L, P<0.001) was much lower than that before treatment (22.99±4.46 g/L) in respond elderly ITP group, but still higher than that in controls (11.15±4.61 g/L, P<0.05). CD19^+^ cell percentage in respond elderly ITP group was 11.78±4.91%, which was lower than that before treatment (16.46±6.03%, P<0.001) and did not differ significantly from the controls (Table 3).

### IgG and T lymphocyte subsets expression in both ITP and elderly ITP patients (NR) before and after first-line treatment

After treatment, the levels of serum IgG (12.00±5.46 g/L, P<0.001) and CD19^+^ cell percentage (11.01±7.85%, P<0.05) were much lower than that before treatment (19.54±4.28 g/L, 12.47±2.19%, respectively) in non-respond ITP group and did not differ significantly from the controls. CD8^+^ cell percentage in non-respond ITP group was 31.09±7.55% after treatment, which was higher than that in controls (22.99±3.24%, P<0.05). A significant increase in CD4^+^/CD8^+^ cell ratio was seen in non-respond ITP group (1.05±0.33, P<0.05) after treatment when compared with that before treatment (0.98±0.50), but still lower than that in controls (1.56±0.29, P<0.05)(Table 4).

**Table 4:** IgG and T lymphocyte subsets expression in both ITP and elderly ITP patients (NR) before and after first-line treatment(mean±SD).

	IgG(g/L)	CD2(%)	CD3(%)	CD4(%)	CD8(%)	CD4/CD8	CD19(%)	NK(%)
Control	11.15±4.61	73.55±7.46	66.98±6.86	34.79±3.85	22.99±3.24	1.56±0.29	12.47±2.19	14.32±4.28
Before(ITP)	19.54±4.28	71.60±12.90	62.06±13.90	29.51±10.48	32.42±8.07	0.98±0.50	15.68±8.32	12.22±7.46
After(ITP)	12.00±5.46^***^	71.46±11.56	64.59±11.56	31.22±8.46	31.09±7.55^#^	1.05±0.33* ^#^	11.01±7.85*	10.67±6.44
Before(elderly)	20.91±3.95	71.42±11.92	60.57±15.83	29.58±11.53	31.38±8.88	1.01±0.53	15.03±7.91	10.97±7.17
After(elderly)	13.75±5.59^***^	72.66±13.11	64.71±13.15	30.92±9.23	31.82±8.23^#^	1.00±0.28^#^	11.04±9.40	8.79±5.58^#^

*** P<0.001 compared with the group before treatment.

* P<0.05 compared with the group before treatment.

**#** P<0.05 compared with the control group.

After treatment, the level of serum IgG (13.75±5.59 g/L, P<0.001) was much lower than that before treatment (20.91±3.95 g/L) in non-respond elderly ITP group and did not differ significantly from the controls. CD8^+^, NK cell percentage and CD4^+^/CD8^+^ cell ratio in non-respond elderly ITP group after treatment were 31.82±8.23%, 8.79±5.58% and 1.00±0.28 respectively. They did not differ too much compared with those before treatment, but still differed remarkably when compared with the controls (P<0.05)(Table 4).

## Discussion

In this retrospective study, we analyzed absolute lymphocyte counts, IgG levels and T lymphocyte subsets in peripheral blood of 61 adult patients with newly diagnosed ITP before and after treatment in our department. We also studied these items in elderly patients who accounted for a large population and tried to analyze the relationship between these immune parameters and therapeutic outcomes. This might be helpful for clinicians to choose alternative treatments for ITP patients, especially the increasing elderly ones.

In our study, the total response rates for all-age group (72.13%) and for elderly group (63.33%) were similar to that reported before[19–20,23].Decreased peripheral ALC were found in ITP groups with different treatment outcomes and different age groups before treatment. After treatment, ALC in these groups increased and showed no significant differences with the control level, which in turn confirmed an important role of lymphocytes in the pathogenesis of ITP once more. Previous reports discovered that initial lower ALC was a predictor of chronic or persistent ITP in children with acute ITP[24–26]. Hu et al[27] reported that ITP patients with a low ALC at diagnosis had an increased infection. In our study, five patients progressed to chronic ITP and the ALCs were 0.6×10^9^/L, 0.59×10^9^/L, 1.26×10^9^/L, 0.9×10^9^/L, 0.43×10^9^/L in each patient, which was really much off the normal average level. We need to further expand the sample size and look into the relationship between ALC and risk of infection too. Besides, we found that IgG expression and CD19^+^ cell percentage in either whole ITP group or elderly group increased, but CD4^+^/CD8^+^ cell ratio decreased. Although significant differences were observed between ITP patients and controls, there was not much difference between the whole group and elderly group. These results were consistent with what Wei et al[28] reported. These findings further confirmed that both humoral immunity and cellular immunity participated in the pathogenesis of ITP.

According to treatment responses, patients were divided into respond and non-respond groups. Respond group had increased IgG and CD19^+^ cell expression and decreased CD3^+^ cell expression while non-respond group had increased CD19^+^and CD8^+^ cell expression with decreased CD4^+^/CD8^+^ cell ratio in the whole ITP group before treatment. The situation was almost same in elderly ITP patients with different responses. After treatment with IVIG plus corticosteroids, increased IgG and CD19^+^ cell expression were controlled while almost no changes in T cells were found in respond patients. With respect to non-respond patients after therapy, no significant differences were found in IgG and CD19^+^ cell expression compared with the control group. However, CD8^+^ cell percentage was still higher and CD4^+^/CD8^+^ cell ratio was still lower than that in controls. We guess patients with higher levels of CD8^+^ cells and lower levels of CD4^+^/CD8^+^ cell ratio were less likely to respond to first-line treatment. It went well in elderly respond patients while CD8^+^ cell expression and CD4^+^/CD8^+^ cell ratio did not change much after treatment in elderly non-respond patients. These results showed statistical differences when compared with the control group. Furthermore, we unexpectedly discovered that NK cell expression decreased after treatment in elderly non-respond patients and showed statistically difference from that in controls. It seemed that abnormal expression of NK cells made therapies in elderly ITP patients even more difficult.

We comprehensively analyze and interpret the abnormal expression of T lymphocyte subsets and the role of treatment as follows. First, the mechanisms of actions of IVIG are confirmed by many researches[29–33]. On the level of phagocytosis, it can block Fc receptors of the reticuloendothelial system, mainly in the spleen. It down regulates autoantibody production by activated B cells and modulates T-cell reactivity, cytokine release and increases Tregs. Attenuation of complement activation, influence of apoptosis, and modulation of dendritic cells are also involved. Corticosteroids, which inhibit the expression of certain Fc receptors impeding platelet clearance, may also increase platelet production and inhibit antibody production upon prolonged use. Although IVIG has been reported to inhibit the differentiation and amplification of Th17 cells[34] recently and influence T cell activity previously, the combination of IVIG and corticosteroids seems to play a major role in regulating humoral immunity. Seite et al[35] reported IVIG-treated B cells show defects in activating coreceptor expression, calcium signaling, and BCR aggregation on engagement by antigen. Maddur et al[36] indicated that IVIG at therapeutic concentrations could directly inhibit the B cell activation and proliferation through induction of anergy and apoptosis. Several other reports have also highlighted that the regulation of immune responses by IVIG can be indirect on B cells through suppressing the activation and functions of dendritic cells (DC), which can synchronize B cell growth, activation and secretion of immunoglobulins. Looking into our data, it turned out that CD19^+^ B cells were reduced after IVIG plus steroids treatment and so were their produced IgG in both respond and non-respond patients.

Second, the cellular immune pathogenesis of ITP appears to be associated with a Th cell defect which leads to abnormal cytokine secretion. These defects are primarily responsible for directing autoreactive B cells to differentiate into autoantibody secreting plasma cells.CD4^+^ T helper (Th) cells include Th1 cells (producing IFN-γ, IL-2, and TNF-β) and Th2 cells (producing IL-4, IL-5, IL-6, IL-10, and IL-13). Th1 cytokines tend to promote a pro-inflammatory response to facilitate macrophage activation, proliferation of cytotoxic T cells and production of opsonizing antibodies.Th2 responses facilitate B-cell activation and proliferation and promote antibody production. The balance of Th1 and Th2 subsets regulates the immune response[37–38]. Besides Th cells, cytotoxic T cells (CD8^+^ T cells) from ITP patients may have direct lytic effects on platelets. Furthermore, CD3^+^ cells from ITP patients showed increased expression of genes involved in cell-mediated cytotoxicity relative to controls, such as TNF-α, perforin, granzyme A and B[10–12]. In this study, we observed differential expression of CD3^+^, CD8^+^ cells and CD4^+^/CD8^+^ cell ratio relative to controls, which was consistent with data showed before by others. Some of these abnormalities could be corrected while some not according to treatment sensitivity. T cell abnormalities in patients who were not responding could not be corrected by IVIG plus steroids treatment. Thus, it showed tolerance to the first line treatment and might be the reason why the disease became chronic and refractory.Th17 and Tregs are widely investigated recently and reported to be involved in the pathogenesis of many autoimmune diseases including ITP. Maddur et al[39–40] presented that various IVIG preparations with respect to various parameters including stabilizing agents, formulation, source of plasma and IgG purification methods equally inhibited Th17 cell proliferation and IL-17 secretion, which was Fab dependent. In vitro and in vivo studies in experimental models have demonstrated that IVIG can expand Tregs in a cyclooxygenase-2(COX-2)-dependent induction of prostaglandin E2 (PGE2) way in human dendritic cells (DCs)[41–43]. Whether IVIG plays a role in this study by influencing the balance between Th17 cells and Tregs needs more investigation.

Third, NK cells appear to be capable of regulating B-cell immunoglobulin production and their defective activity in autoimmune diseases may have an influence on autoantibody production. Although whether NK cells participate in the pathogenesis of ITP and specific mechanisms underlying the disease need to be further investigated, Johansson et al[44] discovered that prednisolone inhibited antigen-specific NKT cell expansion in ITP patients in remission. Untreated ITP patients also showed reduced NKT cell proliferative capacity, although this was less marked than in treated patients. These results supported a role for NKT cells in ITP. However, our results were somewhat opposite to Johansson’s. In our study, lower numbers of NK cells were found in non-respond ITP patients especially in elderly patients after treatment. Since Jacobi et al[45] found IVIG therapy could reduce the percentage of NK cells and activity of NK cells in the peripheral blood by degranulation of granzyme B and perforin, we guess additional IVIG treatment beside steroids and different effects of drugs on NK cell activity apart from NK cell numbers might contribute to these different results. Semple et al[46] reported that there was a direct correlation between the duration of therapy and NK activity in ITP patients and suppressed NK activity can be rescued by therapy. Karpovitch et al[47] found prednisone treatment did not significantly modify the function and expression of multidrug resistance-1 (MDR1), which is a well-recognized mechanism of chemoresistance, in T and NK cells of ITP patients. Further studies should focus on the effects of IVIG or corticosteroids therapy on NK cell expression or even NK cell activities. MRD1 might be another point we should concern too.

Recently, IVIG sialylation has been reported to play an important role in IVIG anti-inflammation response. However, results of the exact mechanisms are different and it still remains a challenge to the scientists. The mechanisms of action of IVIG involve a wide spectrum of Fab-mediated and, probably, distinct Fc-mediated mechanisms, that may or may not depend on IVIG sialylation[48–49]. Schwab et al[50] discovered a sialic acid and SIGNR1-dependent pathway was responsible for IVIG-mediated suppression of autoantibody-dependent platelet depletion in ITP mice. However, using another antibody-mediated experimental model of ITP, Leontyev et al[51] showed that the protective effect of IVIG was independent of sialylation of IgG. Moreover, use of IVIG enriched for Fab-sialylated IgG resulted in a decrease rather than an increase of the efficacy of IVIG in the used murine model of passive immune thrombocytopenia[52]. In conclusion, it is likely that no single paradigm can entirely account for the mechanism of action of IVIG, even in a given disease model.

More and more studies have now found that the incidence of ITP increases with age[53–54]. In elderly patients over 60 years of age with ITP, most have considerable comorbidities, more common major hemorrhagic events, high mortality directly associated with disease, chronic disease course and poor response to various treatment options[54–56]. In this study, almost half of the patients (30/61) were over 60 years of age and more than half (16/30) were those over 70 years of age. Common comorbidities observed in our elderly patients were hypertension, diabetes, anemia and coronary heart disease. A number of old patients (10%) were asymptomatic despite persistent thrombocytopenia. The remaining 90% patients showed signs of bleeding. This rate was higher than those described in the published literature for the all-age groups, but was similar to that in other series of elderly patients[20,57]. Although severe bleeding events, especially life-threatening ones, are much less common[58–59], in our research, among five patients who became chronic, three were elderly patients and the one died of cerebral hemorrhage was 67 years of age. In these patients, IgG, CD19^+^ and CD8^+^ cell expressions were higher than controls before treatment. After treatment, IgG level and CD19^+^ cell expression were reduced but not CD8^+^ cells. Then, with the ever increasing aging population, attention to the treatment of elderly ITP patients in clinical practice becomes much more important.

Besides, our study had several limitations. First, the corticosteroids treatment varied according to each individual’s clinical condition. It is therefore difficult to analyze the results according to different steroids dosage and treatment duration. Second, it is better to measure the expression of specific anti-platelet antibodies and analyze the relationship between these antibodies and therapeutic effects. Third, not all patients completed bone marrow examination and we didn’t analyze the relationship between megakaryocyte numbers and the treatment responses. Fourth, since this is a retrospective study, Th cytokines in the plasma were not detected as routine examination in our hospital. Hence, we still have a lot of work to do in the near future. Because of the short number of patients investigated, our data should be considered preliminary, awaiting further confirmatory studies.

In conclusion, our study showed that the detection of T lymphocyte subsets might be helpful for the diagnosis and determination of therapeutic outcomes of ITP patients. The levels of CD8^+^ and NKT lymphocytes might be helpful prognostic markers in ITP patients especially in elderly ones. The aberrant immunocyte subsets might be related to complicated pathogenesis of ITP, and further researches are needed to confirm these findings with larger patient numbers and to fully investigate different treatment options responsible for these complicated mechanisms. These results may provide evidence for individualized clinical treatment and finally benefit ITP patients.

## Supporting Information

S1 STROBE Checklist Checklist of items included in this retrospective study. (PDF)
